# Days of Antibiotic Spectrum Coverage (DASC) as a Metric for Evaluating the Impact of Prospective Audit and Feedback (PAF) against Long-Term Broad-Spectrum Antibiotic Use

**DOI:** 10.3390/antibiotics13090804

**Published:** 2024-08-25

**Authors:** Yuichi Shibata, Jun Hirai, Nobuaki Mori, Nobuhiro Asai, Mao Hagihara, Hiroshige Mikamo

**Affiliations:** 1 Department of Pharmacy, Aichi Medical University Hospital, Nagakute 480-1195, Aichi, Japan; shibata.yuuichi.414@mail.aichi-med-u.ac.jp; 2 Department of Infection Control and Prevention, Aichi Medical University Hospital, Nagakute 480-1195, Aichi, Japan; hirai.jun.326@mail.aichi-med-u.ac.jp (J.H.); mori.nobuaki.148@mail.aichi-med-u.ac.jp (N.M.); asai.nobuhiro.039@mail.aichi-med-u.ac.jp (N.A.); 3 Department of Clinical Infectious Diseases, Aichi Medical University Hospital, Nagakute 480-1195, Aichi, Japan; 4 Department of Molecular Epidemiology and Biomedical Sciences, Aichi Medical University, Nagakute 480-1195, Aichi, Japan; hagimao@aichi-med-u.ac.jp

**Keywords:** days of therapy (DOT), days of antibiotic spectrum coverage (DASC), antimicrobial stewardship programs (ASPs), prospective audit and feedback (PAF)

## Abstract

The present study aimed to evaluate the impact of prospective audit and feedback (PAF) on the use of inpatient broad-spectrum antibiotics for more than 10 days using days of therapy (DOT) and a novel metric called days of antibiotic spectrum coverage (DASC) to assess whether the antimicrobial spectrum was narrowed. Conducted at Aichi Medical University Hospital in Japan, the study compared a six-month baseline period (April to September 2022) with a six-month intervention period (April to September 2023). The primary outcome measures were changes in DOT/patient and DASC/patient for broad-spectrum antibiotics. Propensity score matching was performed between two periods and a total of 172 patients were included in the study (pre-intervention, n = 86; intervention, n = 86). The DASC/patient of broad-spectrum antibiotics was statistically decreased in the intervention period compared to that in the baseline period (153.3 vs. 122.7, *p* < 0.05). Additionally, our PAF intervention led to a switch to narrow-spectrum antimicrobial therapy without increasing all-cause 30-day mortality (5.8% vs. 5.8%, *p* = 1.0). However, the DOT/patient, DASC/patient, and DASC/DOT of all antimicrobials were not significantly changed. Our study concluded that we should reconsider the timing of PAF intervention by evaluating the effort of PAF by using DOT and DASC.

## 1. Introduction

Antimicrobial resistance (AMR), defined as the resistance of microbes to drugs that inhibit their growth or cause death [1,2], leads to increased mortality and healthcare costs [3,4]. Since AMR is primarily caused by inappropriate antibiotic use [5], the Infectious Diseases Society of America and Society for Healthcare Epidemiology of America have encouraged the implementation of antimicrobial stewardship programs (ASPs) to prevent unnecessary broad-spectrum antimicrobial use [6]. ASPs, including prospective audit and feedback (PAF), formulary restrictions, and prior authorization, can effectively reduce the use of broad-spectrum antibiotics compared to the pre-intervention phase [7,8,9,10]. 

Days of therapy (DOT) is a critical metric in healthcare, particularly in ASPs, which is used to monitor the duration and frequency of antibiotic use to combat AMR [11]. By curbing unnecessary antibiotic use, DOT helps healthcare providers optimize treatment regimens, ensuring accurate dosing and duration to prevent the emergence of AMR. This optimization not only improves patient outcomes but also generates cost savings by minimizing unnecessary prescriptions and associated complications, ultimately benefiting both healthcare facilities and patients. However, PAF efforts to avoid broad-spectrum antimicrobials are not accurately measured by DOT, because DOT does not consider the antibiotic spectrum and cannot measure efforts to avoid unnecessary broad-spectrum antibiotic use in both empiric and de-escalation therapies [12]. DOT only counts the number of individual antimicrobials per day without considering their spectrum. For instance, if a patient is prescribed a combination of two or more types of antimicrobials, the DOT could be the same while the antimicrobial spectrum is broader (e.g., meropenem and vancomycin vs. ceftriaxone and metronidazole). To overcome the limitation of DOT, Kakiuchi et al. proposed a new metric for antimicrobial consumption: days of antibiotic spectrum coverage (DASC) [13]. DASC is calculated by multiplying DOT and the antibiotic spectrum coverage (ASC) score, which is a summative score of the antimicrobial spectrum [13]. Although several studies have shown that PAF reduces the DOT of long-term used antibiotics [14,15], investigating both the antimicrobial spectrum and volume in PAF is important to more effectively decrease AMR.

The purpose of this study was to evaluate PAF using DASC for the long-term use of broad-spectrum antibiotics and to assess whether the antimicrobial spectrum is narrowed in addition to reducing the DOT of broad-spectrum antibiotics.

## 2. Results

**Figure 1:**
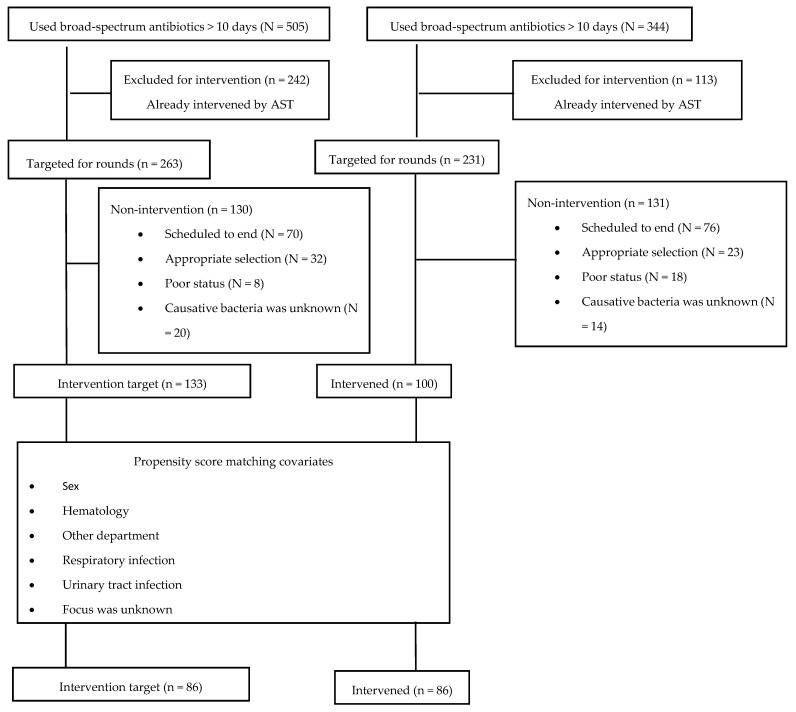
Breakdown of patients in this study. Scheduled to end: discontinuation or change of the target antibiotics by the attending physician. Appropriate selection: selected target antibiotic for detected bacteria and infected organ is appropriate. Poor status: the patient is in the terminal stage, and antimicrobial change is difficult. Causative bacteria was unknown: no ordered cultures nor identified infection source by the antimicrobial stewardship team. AST, antimicrobial stewardship team.

During the study period, the number of patients using broad-spectrum antibiotics for more than 10 days was 505 in the baseline period and 344 in the intervention period (Figure 1). Among these patients, 242 in the baseline period and 113 in the intervention period had already been intervened upon by the AST. In the baseline and intervention periods, 130 and 131 patients, respectively, were excluded from intervention for the following reasons: planned termination, appropriate selection, poor status, and unknown causative bacteria. Furthermore, we performed propensity score matching to adjust for patient backgrounds. Ultimately, 172 patients (baseline period, n = 86; intervention period, n = 86) were included in the study.

Broad-spectrum antibiotic use for more than 10 days with AST intervention and outcomes based on AST recommendations are shown in Table 1. The most common antibiotics requiring intervention were tazobactam/piperacillin (TAZ/PIPC) (47.0%) and carbapenems (37.0%). The rate of change recommendations (53.0%) was higher than discontinuation recommendations (33.0%). The overall acceptance rate of AST recommendations was 86.0% (86/100).

The clinical characteristics of patients administered broad-spectrum antibiotics for more than 10 days are shown in Table 2. After the propensity score matching, 86 patients were selected from each period, and the backgrounds were well-balanced between the two matched periods. Hematology was the most common hospital department targeted for intervention in both periods (29.1% vs. 25.6%, *p* = 0.61). The most common types of infection in both periods were respiratory infections, such as empyema and obstructive pneumonia (27.9% vs. 32.6%, *p* = 0.51).

Changes in primary and secondary outcomes after propensity score matching analysis are shown in Table 3. The DASC/patient (153.3 vs. 122.7, *p* < 0.05) of broad-spectrum antibiotics was significantly decreased after intervention. The antibiotic with the highest rate of decrease was carbapenem (DOT/patient, 31.7%; DASC/patient, 31.6%; *p* < 0.05). The DOT/patient, DASC/patient, and DASC/DOT of all antimicrobials administered from the start to the end of treatment were not significantly changed.

For secondary outcomes, the price/patient for all antibiotics administered from the start to the end of treatment (42,366.1 vs. 43,687.4, *p* = 1.0) and total of broad-spectrum antibiotics (37,929.4 vs. 35,759.2, *p* = 0.82) did not decrease with the intervention. The number of *Clostridioides difficile* infection (CDI) tests (26.22 vs. 26.60, *p* = 0.60) remained unchanged before and after the intervention, and the incidence of CDI was not significantly different (1.2% vs. 4.7%, *p* = 0.18). There was no significant difference in 30-day all-cause mortality between the baseline and intervention periods (5.8% vs. 5.8%, *p* =1.0).

**Figure 2:**
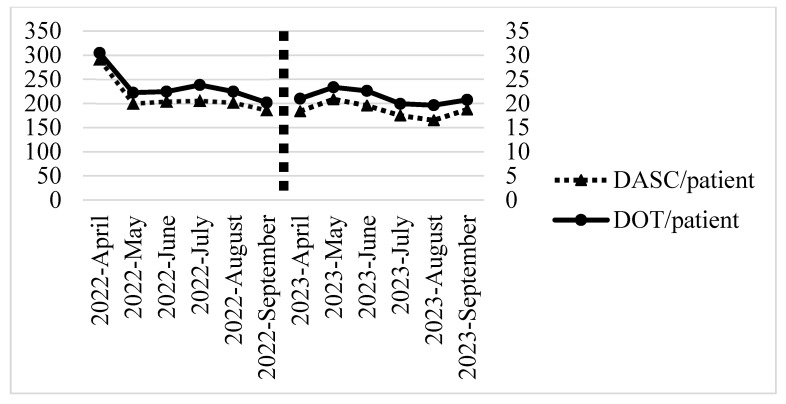
Monthly DOT/patient and DASC/patient of all antibiotics administered from the start to the end of treatment trends during the baseline and intervention periods. DOT, days of therapy; DASC, days of antibiotic spectrum coverage.

**Figure 3:**
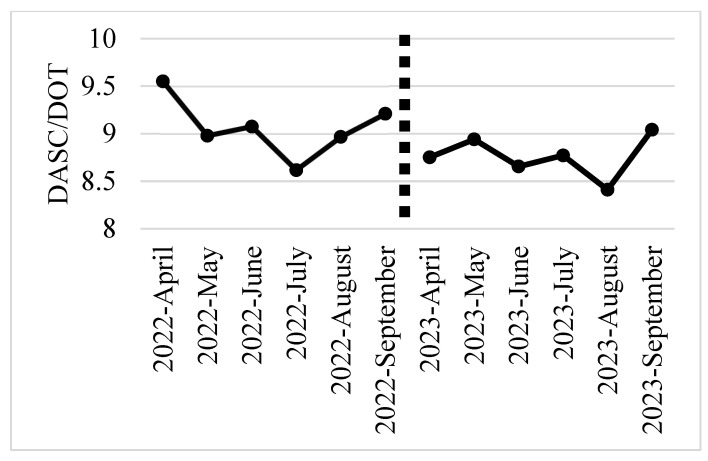
Monthly DASC/DOT of all antibiotics administered from the start to the end of treatment trends during the baseline and intervention periods. DOT, days of therapy; DASC, days of antibiotic spectrum coverage.

Figure 2 and Figure 3 show the monthly trends in the DOT/patient, DASC/patient, and DASC/DOT for all antibiotics administered from the start to the end of treatment during the baseline and intervention periods. Both the DASC/patient and DASC/DOT showed a decreasing trend starting from the first month of the intervention period.

## 3. Discussion

In this study, we evaluated the impact of PAF on inpatient broad-spectrum antibiotic use for more than 10 days using DOT and DASC. We found that the DASC/patient of broad-spectrum antibiotic use was significantly reduced. Additionally, all-cause 30-day mortality did not statistically increase between the baseline and intervention periods despite there being no difference in patient backgrounds between the two periods. However, the DOT/patient, DASC/patient, and DASC/DOT of all antimicrobials administered from the start to the end of treatment were not significantly changed.

Kakiuchi et al. developed DASC as a replacement for DOT for more accurate benchmarking in AS. They conducted a retrospective cohort study comparing the DOT and DASC of antibiotics administered to all patients admitted to acute care units in 124 hospitals between 1 January and 31 December 2018 [13]. They found little correlation between the DOT/patient and DASC/DOT, suggesting that lower antibiotic consumption in a hospital does not necessarily indicate a more frequent use of narrow-spectrum antibiotics. Therefore, they proposed using DASC in AS as a potential replacement for DOT [13]. Conversely, Kanda et al. reported in their Japanese national survey of antimicrobial consumption for inpatients (N = 26,301,685) between 2014 and 2018 that it is difficult to assess the trend in the antibiotic spectrum using DASC alone [16]. They examined the relationship between the trend in DASC and DOT and found that DASC/DOT did not change, although DOT and DASC increased significantly, indicating that the antibiotic spectrum remained constant during the study period. They emphasized that using DASC and the combination of DOT as a quantity indicator and DASC/DOT as a spectrum indicator may allow for more appropriate evaluation in AS [16]. In the present study, we evaluated the effect of PAF using both DASC and DOT for broad-spectrum antibiotics used for more than 10 days and found that the DASC/patient of broad-spectrum antibiotic use showed significant reductions. This result was expected to have been influenced by our intervention. On the other hand, the DOT/patient, DASC/patient, and DASC/DOT of all antimicrobials were not significantly changed. Hence, the DASC/patient of broad-spectrum antibiotics was decreased, but the spectrum of all antibiotics was not changed between the two periods. We assume that this was because the timing of our intervention was late. The major reason for non-intervention was the scheduled end of the broad-spectrum antibiotic use in both groups (Figure 1). Although there was no significant difference, the reduction rate after the intervention of the DASC/patient and DASC/DOT ratios was higher than that of DOT/patient of all antimicrobials. Shortening the timing of the PAF intervention might have reduced the DASC for all antimicrobials. We consider using both DASC and DOT to be essential for evaluating PAF. Further larger studies are needed to assess whether benchmarking using DASC in addition to DOT will decrease long-term broad-spectrum antibiotics used in PAF efforts.

Several recent systematic reviews and meta-analyses have shown that active AS intervention for appropriate treatment is associated with reduced antibiotic consumption, costs, and mortality [17]. However, in the present study, there was no difference in the total price of target antimicrobials between the baseline and intervention periods, even though the DASC of the targeted antibiotics and the DOT/patient for carbapenems had decreased. We assume that this was because the patients in this study had infections requiring long-term antimicrobial therapy, such as empyema and abscesses. Indeed, more than half of the patients in our intervention could not discontinue antimicrobial agents and continued therapy with a switch to narrow-spectrum antimicrobials. We also suspect that the lack of a difference in the incidence of CDI between the two cohorts was influenced by the background of patients requiring long-term antimicrobial therapy. Reducing the long-term use of broad-spectrum antimicrobials without worsening prognosis is an important issue in AS to control widespread antimicrobial resistance. In this study, using DASC, we demonstrated a reduction in the DASC/patient of broad-spectrum antimicrobial use without worsening 30-day all-cause mortality despite there being no difference in terms of patient background between the two periods.

This study had some limitations. First, although our study is the first to evaluate PAF against inpatient broad-spectrum antibiotic use using DASC, it was a single-center retrospective study with a short intervention period, and our results may not be generalizable to other geographical areas with different treatment standards and epidemiological characteristics. Second, in 2023, there was a shortage in meropenem in Japan, and many hospitals in Japan restricted its use. However, our hospital had a stable supply of meropenem, and its use was not restricted. Therefore, we propose that the decrease in DASC for carbapenem was affected by the PAF intervention, and not by the meropenem shortage.

## 4. Materials and Methods

### 4.1. Study Setting and Design

This retrospective study was conducted at the Aichi Medical University Hospital (AMUH), a tertiary care hospital in Japan with 900 inpatient beds, including a 40-bed intensive care unit. We compared the DASC in the PAF program for long-term broad-spectrum antibiotics use between a six-month baseline period (April to September 2022) and a six-month intervention period (April to September 2023). Data were obtained from the AMUH medical database, including microorganism data from the microbiology laboratory, prescription data from the pharmacy department, patient data from the AS team (AST), and chart reviews. The JAID/JSC Guide for Management of Infectious Diseases, the guideline for infectious disease in Japan, was not changed between the two periods.

### 4.2. Ethics

The study was approved by the Research Ethics Committee of Aichi Medical University (approval number 2023-683). Patients’ anonymity and privacy were protected. Informed consent was not required due to the retrospective nature of this study. An online opt-out option was clearly described and made available to all patients.

### 4.3. Intervention

The AST consisted of two infectious disease physicians and one pharmacist dedicated to the AS program. PAF was implemented for inpatient broad-spectrum antibiotic use exceeding 10 days starting in April 2023. The 10-day threshold was set due to resource constraints, as infectious disease physicians also provided consultations, interventions for patients with bacteremia or resistant bacteria, outpatient services, and infection control. Broad-spectrum antibiotics included carbapenems, anti-methicillin-resistant *Staphylococcus aureus* (MRSA) agents, fluoroquinolones, tazobactam/piperacillin, tazobactam/ceftolozane, colistin, and tigecycline (listed in Table S1). Prior authorization for these broad-spectrum target drugs was not required at AMUH. PAF against broad-spectrum antibiotic use was performed 3 days per week. Patients who had already received intervention by the AST were excluded. The AST discussed the dose, duration, and appropriateness of the prescribed antibiotics. They recommended that the attending physician discontinue or change the antibiotic if deemed inappropriate by the AST. If the same patients were treated during different periods, they were counted as separate patients.

### 4.4. Propensity Score Matching

The propensity score matching was calculated using the logistic regression model to balance the baseline characteristics and potential confounders between the two periods. In this study, patients were matched one-to-one by the propensity score using the covariates of sex, the percentage of hematology, other departments, respiratory infection, urinary tract infection, and focus was unknow as the confounding variables.

### 4.5. Outcome Measures

The primary outcomes were changes in DOT per patient and DASC per patient for broad-spectrum antibiotics. We also compared the DOT/patient and DASC/patient for all antimicrobials administered from the start to the end of treatment between the baseline and intervention periods. Secondary outcomes included changes in price per patient, the incidence of CDI, and all-cause 30-day mortality.

DOT/patient was calculated as a volume indicator of antimicrobials provided to hospitalized patients, while DASC/patient was a composite measure of the volume and spectrum of antimicrobials prescribed. DASC was calculated using the ASC score defined in a previous study [13]. The ASC score is a system used to evaluate the spectrum of antibiotics. The score categorizes clinically important bacterial pathogens into two domains: wild-type organisms without acquired resistance and commonly isolated microorganisms with specific mechanisms of acquired resistance. The wild-type category includes 11 organism groups, whereas the acquired resistance category includes 5. The ASC score was added based on the number of activities of each antibiotic against the 16 categorized strains. If an antibiotic was used that was not defined by the ASC score, the score of a similar antibiotic class was adopted based on the method of the previous study and discussion between the infectious diseases physicians and the pharmacist in the AST. The price/patient of antibiotics was calculated by converting the price of antibiotics adopted in AMUH during the study period to price/g. The ASCs and price/g for antibiotics are listed in Table S2. DASC/patient indicates the total antibiotic spectrum and dosing period per patient. DOT/patient indicates the dosing period per patient. DASC/DOT indicates the selected ASC score per day. We collected data regarding age, sex, and the CCI to compare the background of patients between the two periods. The CCI was used to evaluate underlying diseases [18].

CDI was defined by the presence of gastrointestinal symptoms (e.g., diarrhea on the Bristol Stool Scale, scores 5–7), a clinical suspicion of CDI, and a positive result for *C. difficile* toxins by rapid immunoenzyme assay for glutamate dehydrogenase (GDH) and a toxin (GE Test Immunochromato-CD GDH/TOX; Nissui Pharmaceutical Co., Ltd., Himeji, Japan) or polymerase chain reaction of the toxin B gene using the Cepheid GeneXpert C difficile Assay (Beckman Coulter Inc., Brea, CA, USA). CDI was monitored for 60 days after the initiation of target antibiotic treatment. The incidence of CDI was expressed as the number of cases per patient enrolled in the study, and testing density was expressed as the number of tests per 10,000 PD. Only tests performed on patients enrolled in the study were used to determine CDI incidence and testing density. Patient-day data were based on all patients admitted to the study units, including those not enrolled in the study. All-cause 30-day mortality was defined as death occurring within 30 days of starting target antibiotics treatment.

The acceptance rate of AST recommendations was defined as the number of recommendations accepted by prescribers divided by the total number of recommendations made. Acceptance or non-acceptance was determined by chart review within 24 h of the recommendations. The AST conducted a chart review and determined that no intervention was required under the following conditions: scheduled to end, appropriate selection, poor status, and unknown causative bacteria. “Scheduled to end” was defined as a discontinuation or change in the broad-spectrum antibiotics by the attending physician. “Appropriate selection” was defined as selecting an antibiotic appropriate for the identified bacteria and infected organs. “Poor status” referred to patients in the terminal stage where an antimicrobial change was difficult. “Unknown causative bacteria” referred to cases without ordered cultures or identified sources of infection by the AST.

### 4.6. Statistical Analysis

Statistical significance was determined using the 2 × 2 chi-square test, and the Fisher test for categorical data, and the Mann–Whitney U test for continuous data. The statistical significance of the Mann–Whitney U test was set at *p* < 0.05. SPSS statistics version 23.0 (IBM Japan, Tokyo, Japan) was used for all statistical analyses.

## 5. Conclusions

The timing of PAF intervention should be reconsidered by evaluating the effort of PAF by using DOT and DASC. Further research is warranted to determine the broader impact of reducing broad-spectrum antimicrobial exposure on clinical outcomes, costs, and AMR detection rates.

## Figures and Tables

**Table 1:** Broad-spectrum antibiotics used for > 10 days with intervention by AST and results according to AST recommendations (n = 100).

Antibiotics Requiring Intervention	Results after Recommendations	n	(%)
TAZ/PIPC (n = 47)	Change	26	(55.3)
	CFPM	7	(14.9)
	SBT/ABPC	7	(14.9)
	CMZ	5	(10.6)
	CTRX	2	(4.3)
	MINO p.o.	2	(4.3)
	CAZ	1	(2.1)
	CEZ	1	(2.1)
	DOXY	1	(2.1)
	Discontinue	18	(38.3)
	TAZ/PIPC continued	3	(6.4)
Carbapenems (n = 37)	Change	23	(62.2)
	CMZ	6	(16.2)
	CFPM	5	(13.5)
	SBT/ABPC	4	(10.8)
	TAZ/PIPC	4	(10.8)
	LVFX p.o.	2	(5.4)
	CFPM + MNZ	1	(2.7)
	CTRX	1	(2.7)
	Discontinue	6	(16.2)
	Carbapenem continued	7	(18.9)
Anti-MRSA agents (n = 9)	Change	3	(33.3)
	DOXY	3	(33.3)
	Discontinue	5	(55.6)
	Anti-MRSA agents continued	1	(11.1)
Fluoroquinolones (n = 7)	Discontinue	4	(57.1)
	Fluoroquinolones continued	3	(42.9)
Total (N = 100)	Change	53	(53.0)
	Discontinue	33	(33.0)
	Acceptance rate of AST recommendations	86	(86.0)

AST, antimicrobial stewardship team; CAZ, ceftazidime; CEZ, cefazoline; CFPM, cefepime; CMZ, cefmetazole; CTRX, ceftriaxone; DOXY, doxycycline; LVFX, levofloxacin; MINO, minocycline; MRSA, methicillin-resistant *Staphylococcus aureus*; SBT/ABPC, sulbactam/ampicillin; TAZ/PIPC, tazobactam/piperacillin.

**Table 2:** Clinical characteristics of patients administered broad-spectrum antibiotics for more than 10 days.

	Pre-Match			Post-Match		
	Baseline Period	Intervention Period	*p*-Value	Baseline Period	Intervention Period	*p*-Value
	(n = 133)	(n = 100)		(n = 86)	(n = 86)	
Age (years), median (IQR)	74.0 (61.0–83.3)	71.0 (59.0–83.3)	1.0 a	73.0 (59.5–81.0)	71.0 (59.3–80.8)	0.98 a
CCI, median (IQR)	2.0 (2.0–4.0)	3.0 (2.0–5.3)	0.46 a	2.0 (2.0–4.0)	3.0 (2.0–5.0)	0.41 a
Sex (Male/Female)	91/42	56/44	0.05 b	51/35	53/33	0.76 b
Department						
Respiratory medicine (n, %)	23 (17.3)	14 (14.0)	0.50 a	12 (14.0)	11 (12.8)	0.82 b
Hematology (n, %)	53 (39.9)	22 (22.0)	<0.05 a	25 (29.1)	22 (25.6)	0.61 b
Vascular surgery (n, %)	13 (9.8)	15 (15.0)	0.23 a	13 (15.1)	15 (17.4)	0.68 b
Gastrointestinal surgery (n, %)	9 (6.8)	9 (9.0)	0.53 a	8 (9.3)	9 (10.5)	0.80 b
Hepatobiliary pancreas (n, %)	6 (4.5)	7 (7.0)	0.41 a	6 (7.0)	7 (8.1)	0.77 b
Gastroenterology (n, %)	6 (4.5)	4 (4.0)	0.56 b	4 (4.7)	3 (3.5)	0.50 c
Nephrology (n, %)	4 (3.0)	4 (4.0)	0.47 b	2 (2.3)	5 (5.8)	0.22 c
Others (n, %)	18 (13.5)	25 (25.0)	<0.05 a	16 (18.6)	19 (22.1)	0.57 b
Infection type						
Respiratory infection (n, %)	64 (43.6)	31 (31.0)	0.05 a	24 (27.9)	28 (32.6)	0.51 b
Intra-abdominal infection (n, %)	21 (15.8)	17 (17.0)	0.80 a	19 (22.1)	17 (19.8)	0.71 b
Febrile neutropenia (n, %)	26 (19.6)	18 (18.0)	0.77 a	19 (22.1)	16 (18.6)	0.57 b
Skin and soft tissue infection (n, %)	22 (16.5)	19 (19.0)	0.63 a	21 (24.4)	20 (23.3)	0.86 b
Urinary tract infection (n, %)	4 (3.0)	9 (9.0)	<0.05 a	1 (1.2)	1 (1.2)	0.75 c
Focus was unknown (n, %)	3 (2.3)	5 (5.0)	0.22 b	3 (3.5)	3 (3.5)	0.66 c
Others Infections (n, %)	8 (6.0)	6 (6.0)	1.0 a	3 (3.5)	4 (4.7)	0.50 c

Infectious types included duplicate patients. a, Mann–Whitney U test; b, 2 × 2 chi-square test; c, Fisher’s test. CCI, Charlson Comorbidity Index.

**Table 3:** Comparison of primary and secondary outcomes between baseline and post-intervention periods after propensity score matching analysis.

	Baseline Period	Intervention Period	*p*-Value
	(n = 86)	(n = 86)	
**Primary outcome**			
DOT/patient			
Broad-spectrum antibiotics			
TAZ/PIPC	3.84	3.72	0.82 a
Carbapenems	5.15	3.52	<0.05 a
Anti-MRSA agents	2.24	2.16	1.0 a
Fluoroquinolones	3.06	2.44	0.09 a
TAZ/CTLZ	0	0.03	0.70 a
Total of broad-spectrum antibiotics	14.3	11.9	0.09 a
All antimicrobials administered from treatment start to end	23.4	21.3	0.24 a
DASC/patient			
Broad-spectrum antibiotics			
TAZ/PIPC	42.2	40.9	0.82 a
Carbapenems	61.8	42.3	<0.05 a
Anti-MRSA agents	12.0	11.6	1.0 a
Fluoroquinolones	37.3	27.7	0.07 a
TAZ/CTLZ	0	0.28	0.70 a
Total of broad-spectrum antibiotics	153.3	122.7	<0.05 a
All antimicrobials administered from treatment start to end	211.6	186.6	0.09 a
DASC/DOT			
All antimicrobials administered from treatment start to end	9.05	8.76	0.09 a
**Secondary outcome**			
Price/patient			
Broad-spectrum antibiotics			
TAZ/PIPC	11,007.8	12,152.2	0.49 a
Carbapenems	12,938.6	9580.9	0.09 a
Anti-MRSA agents	13,458.9	13,333.7	1.00 a
Fluoroquinolones	524.0	478.3	0.39 a
TAZ/CTLZ	0	214.1	0.70 a
Total of broad-spectrum antibiotics	37,929.4	35,759.2	0.82 a
All antimicrobials administered from treatment start to end	42,366.1	43,687.4	1.0 a
CDI			
Incidence of CDI (n, %)	1 (1.2)	4 (4.7)	0.18 b
Number of CDI tests (10,000 patient days)	26.22	26.6	0.60 a
All-cause 30-day mortality (n, %)	5 (5.8)	5 (5.8)	1.0 c

CDI, *Clostridioides difficile* infection; DASC, days of antibiotic spectrum coverage; DOT, days of therapy; MRSA, methicillin-resistant *Staphylococcus aureus*; TAZ/CTLZ, tazobactam/ceftolozane; TAZ/PIPC, tazobactam/piperacillin. a, Mann–Whitney U test; b, Fisher’s test; c, 2 × 2 chi-square test.

## Data Availability

The original contributions presented in the study are included in the article/Supplementary Materials; further inquiries can be directed to the corresponding authors.

## Supplementary Materials

The following supporting information can be downloaded at https://www.mdpi.com/article/10.3390/antibiotics13090804/s1: Table S1. Types of target antibiotics; Table S2. ASC scores and price of antibiotics.
