# Supplementary material for: Days of Antibiotic Spectrum Coverage (DASC) as a Metric for Evaluating the Impact of Prospective Audit and Feedback (PAF) against Long-Term Broad-Spectrum Antibiotic Use
Source: Antibiotics (Basel). 2024 Aug 25;13(9):804. doi: 10.3390/antibiotics13090804 (PMC11428810; doi:10.3390/antibiotics13090804)
Supplement: Supplementary file 1 [file antibiotics-13-00804-s001.zip › antibiotics-3072808-supplementary.pdf]

Supplemental data

Table S1. Types of target antibiotics.

Anti-MRSA agents	Vancomycin
	Teicoplanin
	Arbekacin
	Linezolid
	Tedizolid
	Daptomycin
Carbapenems	Imipenem/cilastatin
	Meropenem
	Doripenem
	Imipenem/cilastatin/relebactam
Fluoroquinolones	Ciprofloxacin
	Pazufloxacin
	Levofloxacin
	Moxifloxacin
	Sitafloxacin

	Garenoxacin
	Lascufloxacin
Penicillin	Tazobactam/Piperacillin
Cephalosporin	Tazobactam/Ceftolozane
Others	Colistin
	Tigecycline

Vancomycin was monitored by injection route only.

Linezolid, tedizolid, and fluoroquinolones were monitored both orally and parenterally.

Table S2. ASC scores and price of antibiotics.

ASC score	Antibiotics	Price (¥/g)
1	None	
2	Metronidazole (i.v.)	2376.0
	Metronidazole (p.o.)	144.8
3	Penicillin G	126.0
	Cefazolin	346.0
	Cephalexin	291.0
	Erythromycin	59.0
4	Aztreonam	1091.0
	Cefdinir	64.4
	Cefteram	424.0
	Cefotiam	373.0
	Cefcapene	411.0
	Cefditoren	566.0
	Cefuroxime	248.0
	Cefpodoxime	472.0
5	Teicoplanin	7225.0
	Rifampin	112.7
	Amoxicillin	40.4
	Ampicillin	481.0
	Clarithromycin	150.0

	Vancomycin	1505.0
	Roxithromycin	193.3
6	Colistin	56093.3
	Linezolid (i.v.)	16440.0
	Linezolid (p.o.)	11151.8
	Tedizolid (i.v.)	127525.0
	Tedizolid (p.o.)	93630.5
	Azithromycin (i.v.)	3908.0
	Azithromycin (p.o.)	635.6
	Daptomycin	25757.1
	Clindamycin (i.v.)	970.0
	Clindamycin (p.o.)	158.7
	Cefotaxime	799.0
	Ceftazidime	686.0
	Ceftriaxone	422.0
7	Amoxicillin/clavulanic (tablet)	182.8
	Amoxicillin/clavulanic (powder)	114.3
	Sulfamethoxazole/trimethoprim (i.v.)	455.0
	Sulfamethoxazole/trimethoprim (p.o.)	69.2
	Doxycycline	220.0
	Cefmetazole	486.0
	Piperacillin	332.0

	Flomoxef	1286.0
8	Arbekacin	28750.0
	Amikacin	3050.0
	Minocycline (i.v.)	2190.0
	Minocycline (p.o.)	280.0
	Fosfomycin (i.v.)	431.5
	Fosfomycin (p.o.)	128.2
	Isepamicin	2575.0
	Dibekacin	5760.0
	Cefepime	522.0
	Ceftolozanet/azobactam	3978.0
	Cefozopran	956.0
	Ampicillin/sulbactam	398.0
9	Sulbactam/cefoperazone	313.0
	Latamoxef	1153.0
	Pazufloxacin	2390.0
	Gentamicin	7275.0
	Ciprofloxacin (i.v.)	2997.5
	Ciprofloxacin (p.o.)	164.5
10	Faropenem	722.0
11	Piperacillin/tazobactam	265.6
12	Doripenem	1944.0

	Imipenem/cilastatin	1990.0
	Meropenem	1138.0
	Levofloxacin (i.v.)	6274.0
	Levofloxacin (p.o.)	266.6
	Garenoxacin	713.0
	Lascufloxacin (i.v.)	26413.3
	Lascufloxacin (p.o.)	3950.7
	Biapenem	4553.3
	Tebipenem	596.2
	Panipenem/betamipron	1754.0
13	Imipenem/cilastatin/relebactam	17957.6
	Moxifloxacin	608.5
14	Sitafloxacin	1966.0
15	Tigecycline	242180.0
16	None	
